# Characterization of the Vaginal Microbiome in Women with Infertility and Its Potential Correlation with Hormone Stimulation during *In Vitro* Fertilization Surgery

**DOI:** 10.1128/mSystems.00450-20

**Published:** 2020-07-14

**Authors:** Changying Zhao, Zefeng Wei, Junjie Yang, Jiaming Zhang, Chunna Yu, Aijun Yang, Min Zhang, Lin Zhang, Ye Wang, Xiaofeng Mu, Xueyuan Heng, Huijun Yang, Zhongtao Gai, Xuenan Wang, Lei Zhang

**Affiliations:** a Shandong Children’s Microbiome Center, Research Institute of Pediatrics, Qilu Children's Hospital, Cheeloo College of Medicine, Shandong University, Jinan, China; b Institute for Medical Dataology, Cheeloo College of Medicine, Shandong University, Jinan, China; c Department of Biostatistics, School of Public Health, Cheeloo College of Medicine, Shandong University, Jinan, China; d Department of Reproductive Medicine, Affiliated Hospital of Jining Medical University, Jining, China; e College of Life Science, Qilu Normal University, Jinan, China; f School of Basic Medical Sciences, Shandong University, Jinan, China; g Qingdao Human Microbiome Center, the Affiliated Central Hospital of Qingdao University, Qingdao, China; h Microbiological Laboratory, Linyi People’s Hospital, Linyi, China; i Maternal and Child Health Care Hospital of Shandong Province, Jinan, China; University of California, San Francisco

**Keywords:** vaginal microbiome, infertility, *in vitro* fertilization, ovulation induction

## Abstract

Perturbation of vaginal microbiome of reproductive-age women influences all the phases of a woman's reproductive life. Although studies have shown that dynamic changes in vaginal microbiome can affect pregnancy, its role in secondary infertility (i.e., inability to become pregnant or to carry a pregnancy successfully after previous success in delivering a child) and *in vitro* fertilization (IVF) remains to be unraveled. To determine the vaginal microbiome in women undergoing *in vitro* fertilization and embryo transfer (IVF-ET) and investigate its potential correlations with hormone stimulation, we recruited 30 patients with secondary infertility and receiving IVF and 92 matched healthy women and analyzed their vaginal microbiome composition using 16S rRNA gene sequencing. Our results show that women suffering from infertility (infertile women) exhibit a significant decrease in microbiome diversity and richness compared with healthy women during the nonovulation period (follicular phase) (*P < *0.01), whereas vaginal microbiome of healthy women reveals dramatic fluctuations during ovulation (*P < *0.05). Interestingly, infertility patients show no change of the vaginal microbiome under conditions of gonadotropin-releasing hormone (GnRH) agonist and recombinant human chorionic gonadotropin (r-hCG) induction (*P > *0.05). Moreover, our results indicate that infertile women show characteristic variations in vaginal microbiome, such as increased abundance *of Atopobium, Aerococcus*, and *Bifidobacterium* and decreased abundance of *Lactobacillus* and *Leuconostoc*.

**IMPORTANCE** The microbiome had been hypothesized to be involved in the physiology and pathophysiology of assisted reproduction before the first success in IVF, while the data supporting or refuting this hypothesis were less than conclusive. Thanks to sequencing data from the 16S rRNA subunit, we characterized the microbiome in the reproductive tract of infertile women, and we found that changes in the vaginal microbiome are related to female infertility. We also found that the characteristic microbiome bacteria are mainly members of several genera and that the vaginal microbiome of infertile women is not sensitive to hormonal changes during IVF. In conclusion, our report provides data that can be used for discovering the role of the vaginal microbiome in patients suffering from secondary infertility.

## Abstract (precis)

The microbiome had been hypothesized to be involved in the physiology and pathophysiology of assisted reproduction before the first success in IVF, while the data supporting or refuting this hypothesis were less than conclusive. Thanks to sequencing data from the 16S rRNA subunit, we characterized the microbiome in the reproductive tract of infertile women, and we found that changes in the vaginal microbiome are related to female infertility. We also found that the characteristic microbiome bacteria are mainly members of several genera and that the vaginal microbiome of infertile women is not sensitive to hormonal changes during IVF. In conclusion, our report provides data that can be used for discovering the role of the vaginal microbiome in patients suffering from secondary infertility.

## INTRODUCTION

Recent global demographic surveys indicated that infertility remains an ongoing reproductive problem (1). Globally, 10% to 15% of couples are infertile and the couples suffering from secondary infertility (i.e., those unable to become pregnant or to carry a pregnancy successfully after previous success in delivering a child) outnumber those suffering from primary infertility (2). In the past decade, the existence of an extensive microbiome in and on the human body became a subject of mainstream scientific research. The symbiotic relationship between the host and the residing microorganism is necessary to maintain health and avoid disease, and an imbalance in this relationship can lead to poor physiological conditions (3, 4). The vaginal microbiome has an established role in female reproductive tract physiology, pathogen defense, and function (5, 6). The complex interaction between the vaginal microbiome and host physiology plays a pivotal biological role in women (7).

Longitudinal analysis has revealed that the vaginal microbiome of the nonpregnant woman is highly dynamic and is influenced by ethnicity, sexual activity, menstrual cycle, and the local microbiota (8, 9). Normal pregnancy is characterized by a type of microbiome community that has low diversity and high stability (10), and pregnancy capability seems to be affected by the female reproductive tract microbiome (11). Studies have shown that alterations in vaginal microbiology are associated with many pathological conditions, including late miscarriage and premature birth (11), and pathogens such as Chlamydia trachomatis, Mycobacterium tuberculosis, and Neisseria gonorrhoeae can lead to infertility (12). It has been indicated that fertility problems may be due to pathogen changes in the microbiome of the female reproductive tract from the vagina to the upper genital tract, local microbial deformation caused by blood transmission of infectious microorganisms, and retrograde spread of the peritoneal cavity (13). Hormones have been suggested to play an important role in this process (14). The vaginal microbiome changes during normal menstrual cycles, with different estrogen levels in the physiological range (15). These indicate that hormonal status determines the complement of the microbiome residing on the vaginal epithelial mucosa and the level of susceptibility to infection.

*In vitro* fertilization (IVF) procedures, which are designed to overcome infertility and produce successful pregnancy, have been around for almost 40 years and are widespread worldwide. It has been suggested that multiple processes of IVF may affect changes in vaginal microbiome (16). However, given there are a large number of unexplained IVF failures, considering the vaginal microbiome and its impact on female fertility is reasonable. A recent study discovered that women with a low percentage of *Lactobacillus* in the vaginal microbiome have a lower rate of success of embryo implantation, and without a favorable microbiome, the implantation and subsequent development of the embryo appear to be compromised (17).

Therefore, the vaginal microbiome may be a cause of female infertility and embryo implantation failure in IVF that cannot be ignored, and we suspect that these vaginal microbiome changes are related to hormonal status. In this study, we aimed to reveal the characteristics of the vaginal microbiome in a cohort of Chinese female infertility patients and to analyze variations of the vaginal microbiome in ovulating female and infertility patients after injection of hormones during IVF surgery.

## RESULTS

### Study population.

**FIG 1:**
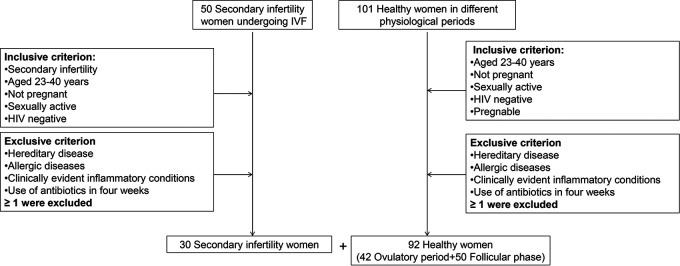
The flow chart of this study.

We enrolled 30 patients with secondary infertility who were to receive IVF surgery and 92 healthy women for the current gut microbiome study (Fig. 1). The characteristics of the patients and the healthy women are detailed in Table 1. Clinical samples were divided into the following four groups: group B-I (patient samples from women suffering from secondary infertility collected during the first 3 days of the follicular phase), group A-I (patient samples from women suffering from secondary infertility collected after gonadotropin-releasing hormone [GnRH] agonist and recombinant human chorionic gonadotropin [r-hCG] administration), group O (samples taken from healthy women during the first 3 days of the ovulation period) and N-O (samples taken from healthy women during 3 days of the nonovulation period [follicular phase]).

**TABLE 1:** Characteristics of study participants^*a*^

Characteristic	Value(s)				*P* value (O vs NO)
	B-I (*n* = 30)	A-I (*n* = 8)	O (*n* = 42)	N-O (*n* = 50)	
Age (yrs)	30.31 ± 6.76	31.12 ± 5.02	31.11 ± 7.05	30.97 ± 7.61	0.7662
Vaginal pH (mean)	NA	NA	4.3	4.1	
No. (%) with VCG I to II	21 (70)	5 (63)	17 (40)	21 (42)	
No. (%) with VCG III to IV	9 (30)	3 (37)	25 (60)	29 (58)	

a Abbreviations: B-I, infertile women before ovulation induction; A-I, infertile women after ovulation induction; O, ovulation-phase healthy women; N-O, follicular-phase healthy women; VCG, vaginal cleanliness grade; NA, not applicable.

### Infertile women harbor an altered vaginal microbiome compared with healthy controls.

**FIG 2:**
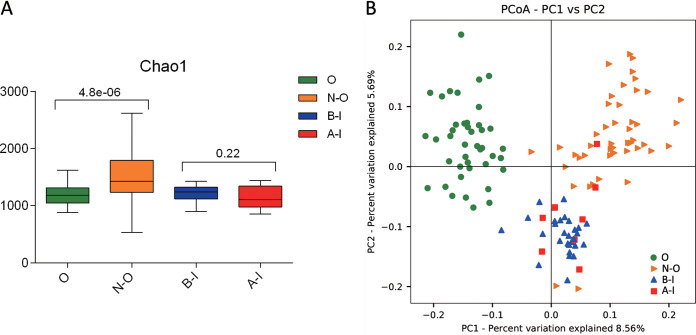
Population statistics of different groups, including groups B-I (infertile women before ovulation induction), A-I (infertile women after ovulation induction), O (ovulatory healthy women), and N-O (follicular-phase healthy women). (A) Comparison of alpha diversity data (Shannon index values) based on the OTU profiles in the O, N-O, B-I, and A-I groups. The *P* value was calculated by the Wilcoxon rank sum test. (B) PCoA of bacterial beta diversity based on the unweighted UniFrac distance and weighted UniFrac distance. O, N-O, B-I, and A-I are colored in green, orange, blue, and red, respectively.

**FIG 3:**
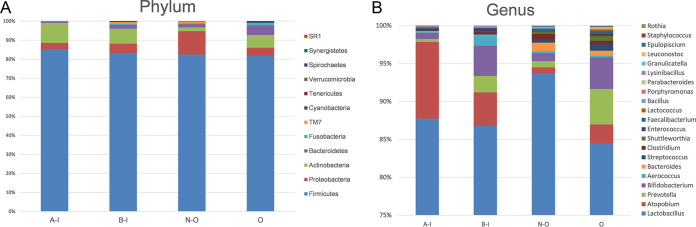
The relative abundances of different taxa at the phylum (A) and genus (B) levels are shown in the bottom panels for the O, N-O, B-I, and A-I groups.

**FIG 4:**
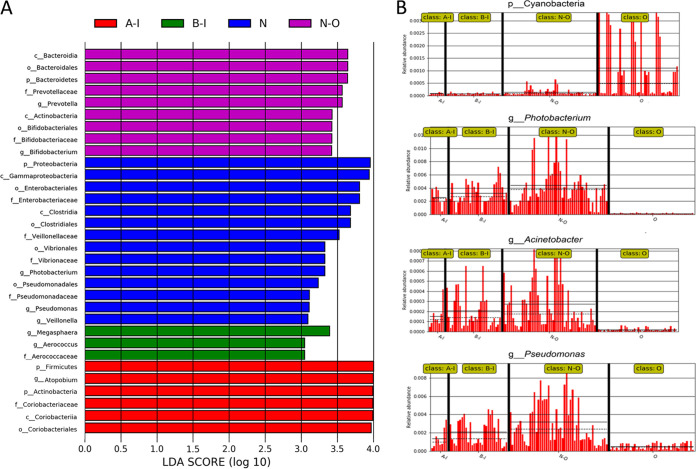
Characteristics of microbial community composition in A-I, B-I, N-O, and O groups. (A) The most differentially abundant taxa between the four groups (LDA score above 3). Data were generated from LEfSe analysis. The LDA score indicates the effect size and ranking of each differentially abundant taxon. (B) Relative abundances of characteristic biomarkers in the four groups.

Analysis of alpha diversity revealed significant differences in Shannon index values between the infertile group and the healthy group during nonovulation (N-O group versus B-I group), representing a marked decrease in microbiome diversity and richness in the B-I group (Fig. 2A, Wilcoxon rank sum test, *P < *0.01). Analysis of beta diversity based on unweighted UniFrac distances revealed that the microbiome of the B-I group was significantly different from that of the N-O group (analysis of similarity [ANOSIM], *r* = 0.1, *P < *0.01, unweighted UniFrac, Fig. 2B). To further explore the features of the vaginal microbiome communities of women suffering from infertility (infertile women), the relative taxon abundances of microbiomes were assessed between the B-I and N-O groups. We conducted abundance analysis at the phylum and genus levels. Among the results, phylum-level analysis demonstrated that the proportion of *Proteobacteria* in the total vaginal microbiome of the patients with infertility was less than that in the total vaginal microbiome of the members of the N-O group and that the proportion of *Actinobacteria* was significantly greater (Fig. 3A). At the genus level, the proportions of *Atopobium*, *Aerococcus*, and *Bifidobacterium* in the total vaginal microbiome of the members of the B-I group were significantly greater than in the N-O group and the proportions of *Lactobacillus* and *Leuconostoc* were slightly lower (Fig. 3B). These significant differences were further confirmed by linear discriminant analysis (LDA) effect size (LEfSe) data, which identified 17 discriminative microbiome signatures (LDA score > 3) that differed significantly in abundance between the B-I and N-O groups (Fig. 4A), such as *Megasphaera*, *Photobacterium*, *Pseudomonas*, *Veillonella*, and *Aerococcus*. All potential biomarkers (LDA score > 2) are shown in Fig. S1 in the supplemental material.

10.1128/mSystems.00450-20.1 FIG S1 Characteristics of microbial community composition in the A-I, B-I, N-O, and O groups. The most differentially abundant taxa between four groups (LDA score > 2) are shown. Download FIG S1, TIF file, 1.9 MB. Copyright © 2020 Zhao et al. 2020 Zhao et al. This content is distributed under the terms of the Creative Commons Attribution 4.0 International license.

### The vaginal microbiome of healthy women shows fluctuations in composition during ovulation.

We compared alpha and beta diversity data between the members of the O and N-O groups, and the results revealed distinct vaginal microbiome compositions. Analysis of alpha diversity showed a significant decrease in microbial diversity (*P < *0.01, Shannon index, Fig. 2A) in the vaginal microbiome of the members of the O group. The compositions of the vaginal microbiome differed in beta diversity between the O and N-O groups based on unweighted UniFrac results (ANOSIM, *r* = 0.143, *P < *0.01, unweighted UniFrac, Fig. 2B). These significant differences were further confirmed by LEfSe analysis, which identified 23 discriminative microbial signatures between the O and N-O groups. Compared with the other groups, the relative abundance of *Cyanobacteria* was increased in the O group, *Bifidobacterium* and *Prevotella* genera were markedly increased in abundance, and the genera of *Photobacterium*, Acinetobacter, *Pseudomonas*, and *Veillonella* were significantly decreased in abundance (Fig. 4A and B). These results showed the differences in the vaginal microbiome between groups O and N-O, which indicated that the vaginal microbiome of women changed during ovulation, and we speculated that this change was related to hormone levels.

### Infertility patients showed no significant changes in the vaginal microbiome under conditions of induction of ovulation in IVF.

To evaluate whether GnRH agonist and r-hCG ovulation induction exerted in IVF surgery has an influence on the vaginal microbiome in infertile women, we compared alpha and beta diversity data between groups B-I and A-I, which revealed that GnRH agonist and r-hCG stimulation of ovulation exerted no influence on the vaginal microbiome in infertile women. Analysis of alpha diversity revealed that the B-I and A-I groups showed no significant difference in Shannon index values (Fig. 2A, Wilcoxon rank sum test, *P > *0.05). No clustering was observed in principal-coordinate analysis (PCoA) before or after GnRH agonist and r-hCG injection (unweighted Unifrac distance, ANOSIM, *P > *0.05, Fig. 2B). We further explored the vaginal microbial community features of infertile women by assessing the relative taxon abundances of microbiome between groups B-I and A-I. At the phylum and genus levels, there were no differences in the classifications of taxa (Fig. 3A and B). However, LEfSe analysis revealed a significant difference in the relative abundances of different bacterial taxa in groups B-I and A-I, among which the levels of the *Veillonella* and *Megasphaera* taxa in the A-I group decreased and that of *Atopobium* increased significantly compared with the B-I group (*P < *0.01, Wilcoxon rank sum test; LDA score > 3.0, Fig. 4A). The results may indicate that although the overall vaginal flora of patients with infertility is not sensitive to hormones, there are still some strains that are more sensitive to hormone therapy during IVF surgery.

### Correlation of candidate bacterial taxa.

**FIG 5:**
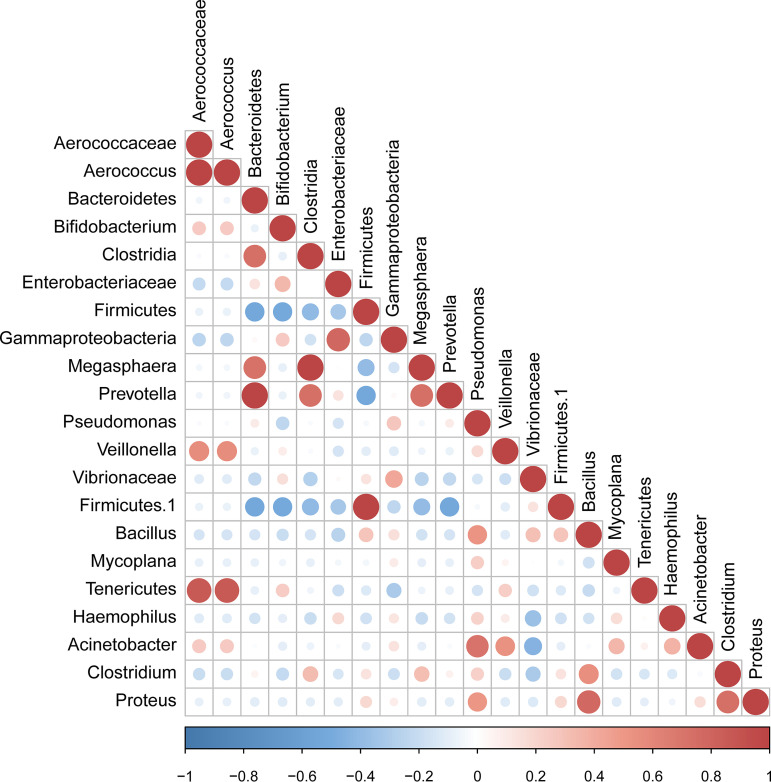
Correlation between candidate taxa. The taxa corresponding to significant differences between the A-I, B-I, N-O, and O groups are indicated (*P* < 0.05, LDA score > 2).

The taxa that met the threshold for significance after modeling were considered candidate bacterial taxa. Correlations between these candidate bacterial taxa were examined (Fig. 5). There were statistically significant positive correlations between the candidate taxa associated with infertility. There were statistically significant positive correlations between the genera *Megasphaera*, *Prevotella*, and *Clostridia*, while *Firmicutes* showed significant negative correlations with *Bacteroidetes and Bifidobacterium*.

## DISCUSSION

This report extends the available knowledge of vaginal microecosystems in female secondary infertility. We compared the vaginal microbiomes of infertile women and healthy women and how they are affected at different menstrual periods. Our study documented that the condition of secondary infertility is accompanied by a characteristic compositional change in the vaginal microbiome.

For women, various endogenous and exogenous factors can alter the relative abundances of *Lactobacillus* and other vaginal microbial components in the vagina (18, 19). Common vaginal microecological disorders include decreases in the levels of of *Lactobacillus* and increases in the levels of other bacteria, most of which are anaerobic bacteria, mainly distributed in the gastrointestinal tract and urogenital tract (20). Studies have shown that *Lactobacillus* can produce lactic acid and short-chain fatty acids, acidify the vaginal environment to a pH level of <4.5, and prevent the growth of other pathogenic bacteria in healthy women (21, 22). We observed an uneven distribution of *Lactobacillus* among the studied cohorts of women and a lower presence of *Lactobacillus* in infertile women than in healthy women in nonovulation (follicular phase). Lactobacilli can act as a barrier against pathogen invasion because their metabolic products secreted in cervical-vaginal fluid are the main causes of differences in bacterial and viral infections (23). Besides, reductions in levels of lactobacilli are associated with an inability to inhibit the colonization of specific harmful microorganisms that increase early abortion rates (24). We believe that the results of this study also generally support the notion that the presence of *Lactobacillus* is beneficial and contributes to maintaining a healthy environment for pregnancy (25).

In our series of assays, although there was no difference between groups A-I and B-I in alpha diversity and beta diversity, we found that the abundances of *Atopobium*, *Aerococcus*, *Megasphaera*, *Prevotella*, and *Bifidobacterium* were increased significantly in infertile women and that the abundance of *Atopobium* was especially increased in the members of the A-I group. *Atopobium* participates in biofilm formation (26), and its presence before antibiotic therapy is associated with partial or complete failure of treatment (27). *Prevotella* and *Megasphaera* are common anaerobic bacterial species in the vagina and are thought to be associated with bacterial vaginosis (BV) (14, 28), and *Aerococcus* is believed to be associated with HIV infection (28). Our results suggest that rather than the synergy of a major species, synergistic effects of different anaerobic bacteria, including *Atopobium*, *Prevotella*, *Bifidobacterium*, and *Megasphaera*, are involved in the pathogenesis of infertility. Decreases in *Lactobacillus* abundance correspond to reductions in lactic acid production, and the metabolic by-products of anaerobic bacteria lead to an increase in normal vaginal pH, which is beneficial to the niche of opportunistic pathogens (29).

Estrogen can stimulate the proliferation of the vaginal epithelium and increase the level of glycogen available in the vagina (30, 31). Therefore, the bacterial composition of the vaginal microbiome can be significantly influenced by the normal fluctuations in estrogen levels that occur during puberty and menopause and in the reproductive period (32–34). And estrogen levels peak during ovulation, so we chose healthy women in nonovulation (follicular phase) and ovulation to study how the ovarian cycle phases—especially ovulation—are linked to distinct vaginal microbiome. However, we found that infertility patients showed no significant fluctuation in vaginal microbiome composition under some conditions of ovulation injection, for example, exposure to gonadotropin-releasing hormone (GnRH), which is involved in the reproductive cycle and regulates the secretion of sex steroids from the gonads. For healthy women, fluctuating levels of both endogenous and exogenous hormones can alter the components of the vaginal microbiome (35). Perhaps differently from healthy women, this hormone-insensitive vaginal flora may be one of the reasons for patients’ infertility.

We observed relatively low levels of *Lactobacillus* during the periovulatory period. Correspondingly, we observed the highest levels of anaerobic bacteria, such as *Prevotella*, *Bifidobacterium*, and *Atopotium*, during this period and found that BV-related bacteria were abundant during the periovulatory period. These results indicated that ovulation might be a period of fragile reproductive tract microecology with respect to disease risk (Fig. 3B; see also Fig. 4A). *Bifidobacterium* bacteria are generally considered to be beneficial members of the intestinal microbiota (36), although their role in the vaginal microbiome has not yet been elucidated. It is conceivable that *Bifidobacterium*, lactic acid-producing bacteria, could have a protective or health-promoting effect in the vagina analogous to that attributed to *Lactobacillus.* Consistent with our results, previous studies have reported an increase in the abundance of *Bifidobacteria* in the reproductive tract of low-abundance *Lactobacillus* (e.g., BV) (28). In addition, a recent study revealed that the presence of a low proportion of *Lactobacillus* in vaginal samples has a negative impact on the success rate of embryo transfer (17).

One of the disadvantages of our study was that the sample size (30 *in vitro* fertilization and embryo transfer [IVF-ET] patients and 92 healthy women) was relatively small. As such, the study might have been underpowered for many statistical tests. However, the use of parametric statistical testing methods which might identify strong effects even with a small sample size compensates for this disadvantage to some extent. The results of this study support the contention that there is an urgent need for large-scale, well-controlled studies of the vaginal microbiome and of IVF-ET outcomes to be designed to further explore the role of the vaginal microbiome in women undergoing *in vitro* fertilization treatment in the future.

## MATERIALS AND METHODS

### Participants and sampling.

Participants aged 23 to 42 years were recruited from the Affiliated Hospital of Jining Medical College (Table 1). The study compared women with secondary infertility to healthy women. Women were eligible to participate if the following inclusion criteria were met: age between 23 and 42 years, not pregnant, HIV negative, no clinically significant treatments within 4 weeks at the start of the study, regular menstrual cycles of 25 to 35 days, no family genetic disease, no clinically obvious inflammation, and no sexual activity within 2 weeks (Fig. 1). Patients enrolled in this study did not have signs or symptoms of cervical, uterine, or tubal infection. The clinicians and the staff members of the embryology laboratory involved in analyses of the IVF cycles followed the appropriate standards, including successive administrations of gonadotropin-releasing hormone (GnRH) agonist in the luteal phase for 6 days, and, once at least one follicle reached a diameter of 18 mm and the diameter of the other two follicles reached 16 mm, 250 μg recombinant human chorionic gonadotropin (r-hCG) was given for 34 to 36 h to induce ovulation. The oocytes were then removed. Swab samples of the posterior fornix of the vagina of patients with secondary infertility were taken twice, once during the first 3 days of the follicular phase before the ovulation period, which were estimated by the menstrual period and examined by B-ultrasound, and once after GnRH agonist and r-hCG administration. Healthy control samples were divided into two groups. One group consisted of samples collected during the follicular period and the other of samples collected during ovulation.

The study was approved by local institutional review boards (Jining, China), and written informed consent was obtained from all patients before they were randomly assigned in a manner that complied with national legislation and the Code of Ethical Principles for Medical Research Involving Human Subjects of the World Medical Association (Declaration of Helsinki). Among a total of 92 healthy women screened, 42 patients were in ovulation, and other 50 were nonovulation (follicular phase). A total of 30 patients with secondary infertility were recruited for and consented to participate in this study.

The vaginal swab samples were collected for sequencing analysis. Samples collected for sequencing analyses were transferred to –80°C storage within 30 min for later analysis.

### Microbial DNA extraction, 16S library preparation, and sequencing.

Microbial DNA was isolated from vaginal swabs using a QIAamp fast DNA stool minikit (Qiagen, Valencia, CA, USA) following the manufacturer instructions. The V1-V2 hypervariable region of the bacterial 16S rRNA gene was identified, to enable analysis of the microbial community within the samples. The following two universal bacterial 16S rRNA gene amplicon PCR primers (PAGE purified) were used: forward primer-27F (5′-AGAGTTTGATCMTGGCTCAG-3′) and reverse primer-355R (5′-GCTGCCTCCCGTAGGAGT-3′) (37).

The PCR was carried out in a 50-μl reaction volume, which included 32.5 μl of double-distilled water (ddH_2_O), 10 μl of 5× HF buffer, 1 μl of 10 mM deoxynucleoside triphosphates (dNTPs), 0.5 μl of 50 mM MgCl_2_, 2 μl each of 10 μM forward and reverse primers, and 1 unit of Phusion DNA polymerase (Thermo Scientific, USA), together with 40 ng of DNA template. PCR was performed under the following conditions: initial denaturation at 98°C for 30 s, followed by 30 cycles of 98°C for 15 s, 66°C for 25 s, and 72°C for 30 s and a final extension at 72°C for 10 min.

The PCR products from the variable regions were processed for parallel tagged sequencing on a HiSeq 2500 platform (Illumina, CA, USA) following previously described procedures (38). Sample-specific barcode sequences were ligated at both ends of the PCR products and quantified using a NanoDrop 2000 spectrophotometer (Thermo Fisher Scientific, USA), and equimolar quantities of each amplified product were pooled. The library pool was then quantified using an Agilent 2100 Bioanalyzer (Agilent Technologies, Inc., USA), followed by amplification and paired-end sequencing on a HiSeq 2500 platform (2 × 250 bp) according to the manufacturer’s instructions.

### Sequence processing and statistical analysis.

The 16S rRNA gene sequence paired-end data set was joined and quality filtered using the FLASH method described previously by Magoč and Salzberg (39). All sequence analyses were provided in the Quantitative Insights into Microbial Ecology (QIIME, version 1.9.1) software suite (40), according to the QIIME tutorial (http://qiime.org/). Chimeric sequences were removed using usearch61 (41) with denovo models. Sequences were clustered against the 97% reference data set of the 2013 Greengenes (13_8 release) ribosomal database (http://greengenes.secondgenome.com). Sequences that did not match any entries in this reference were subsequently clustered into *de novo* operational taxonomic units (OTUs) at 97% similarity with UCLUST. Taxonomy was assigned to all OTUs using the RDP classifier (42) within QIIME and the Greengenes reference data set. Rarefaction and rank abundance curves were calculated from OTU tables using alpha diversity and rank abundance scripts within the QIIME pipeline. Hierarchical clustering based on population profiles of most common and abundant taxa was performed using clustering performed with UPGMA (unweighted pair group method using average linkages) on the distance matrix of OTU abundance. This resulted in a formatted tree, which was obtained utilizing the QIIME package.

### Statistical analysis.

Statistical analyses were carried out in QIIME and in R. To work with normalized data, equal numbers of sequences from all groups were analyzed. The Kruskal-Wallis one-way test of variance was used to compare mean numbers of sequences of the groups, at various taxonomic levels. We performed ANOSIM to analyze the differences in microbial community composition. We applied multivariate association with linear models to find associations of the bacterial interactions. When possible, the analysis provided false-discovery-rate (FDR)-corrected *P* values (FDR values of <0.05 were considered significant for all tests). All data from principal-coordinate analysis (PCoA) were based on unweighted and weighted UniFrac distances calculated using evenly sampled OTU abundances. Linear discriminant analysis effect size (LEfSe) algorithms were employed to find features (taxa) differentially represented between patients and healthy subjects. LEfSe combines the Kruskal-Wallis test or pairwise Wilcoxon rank sum test with linear discriminant analysis (LDA). It ranks features by effect size, placing the features that explain most of the biological difference at the top. Differences were considered significant for *P* values of <0.05.

### Ethical approval and consent to participate.

In this study, which was approved by the Institutional Review Boards of Affiliated Hospital of Jining Medical College (IRB no. AHJ16-0728-01), sample collection began in July 2016. Written informed consent and questionnaire data sheets were obtained from all participants who visited the Affiliated Hospital of Jining Medical College and agreed to serve as sample donors, in compliance with national legislation and the Code of Ethical Principles for Medical Research Involving Human Subjects of the World Medical Association (Declaration of Helsinki).

### Data availability.

All sequencing data associated with this study were uploaded to the NCBI SRA database (accession number PRJNA516352).
