# Supplementary figures and images for: Characterization of the Vaginal Microbiome in Women with Infertility and Its Potential Correlation with Hormone Stimulation during In Vitro Fertilization Surgery
Source: mSystems. 2020 Jul 14;5(4):e00450-20. doi: 10.1128/mSystems.00450-20 (PMC7363005; doi:10.1128/mSystems.00450-20)

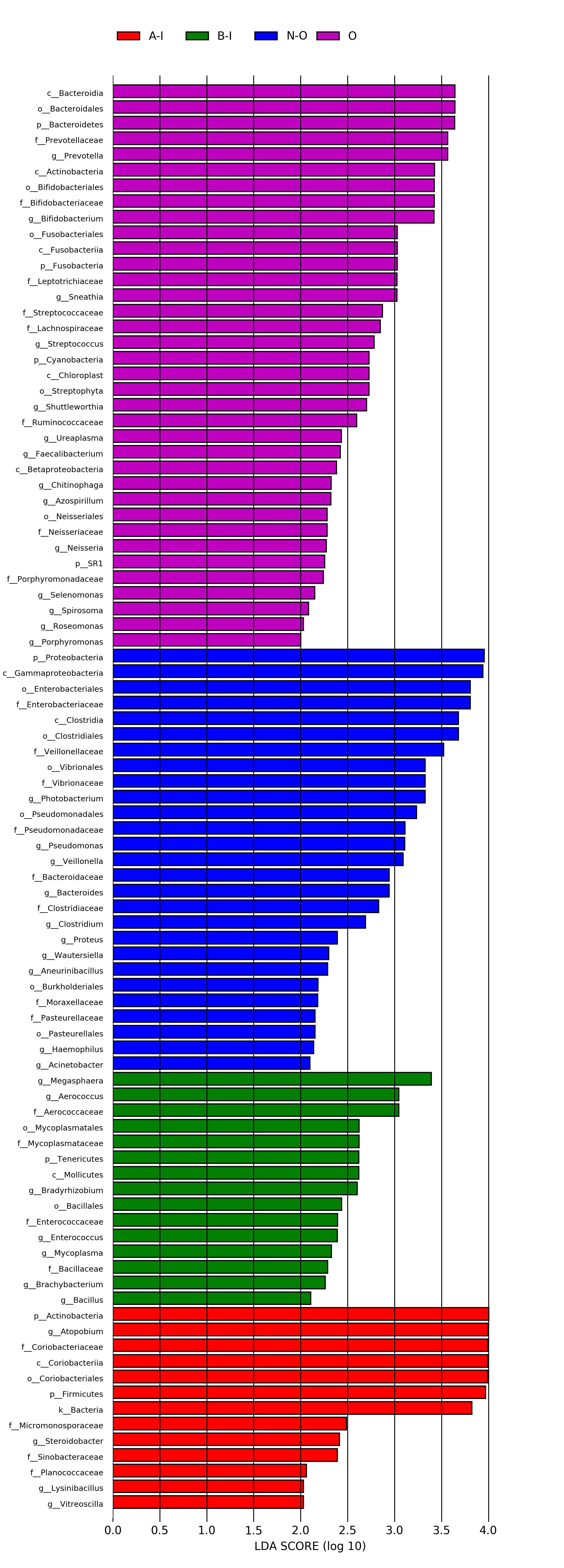

Supplement: FIG S1 [file mSystems.00450-20-sf001.tif]
